# Sex-specific prevalence of coronary heart disease among Tehranian adult population across different glycemic status: Tehran lipid and glucose study, 2008–2011

**DOI:** 10.1186/s12889-020-09595-4

**Published:** 2020-10-06

**Authors:** Seyyed Saeed Moazzeni, Hamidreza Ghafelehbashi, Mitra Hasheminia, Donna Parizadeh, Arash Ghanbarian, Fereidoun Azizi, Farzad Hadaegh

**Affiliations:** 1 Prevention of Metabolic Disorders Research Center, Research Institute for Endocrine Sciences, Shahid Beheshti University of Medical Sciences, P.O. Box: 19395-4763, No. 24, Parvaneh Street, Velenjak, Tehran, Iran; 2 Endocrine Research Center, Research Institute for Endocrine Sciences, Shahid Beheshti University of Medical Sciences, Tehran, Iran

**Keywords:** Coronary heart disease, Diabetes mellitus, Prediabetes, Prevalence, Tehran lipid and glucose study

## Abstract

**Background:**

Coronary heart disease (CHD) is one of the leading causes of death. Alarmingly Iranian populations had a high rank of CHD worldwide. The current study aimed to assess the prevalence of CHD across different glycemic categories.

**Methods:**

This study was conducted on 7718 Tehranian participants (Men = 3427) aged ≥30 years from 2008 to 2011. They were categorized based on glycemic status. The prevalence of CHD was calculated in each group separately. CHD was defined as hospital records adjudicated by an outcome committee. The association of different glycemic categories with CHD was calculated using multivariate logistic regression, compared with normal fasting glucose /normal glucose tolerance (NFG/NGT) group as reference.

**Results:**

The age-standardized prevalence of isolated impaired fasting glucose (iIFG), isolated impaired glucose tolerance (iIGT), both impaired fasting glucose and impaired glucose tolerance (IFG/IGT), newly diagnosed diabetes mellitus (NDM), and known diabetes mellitus (KDM) were 14.30% [95% confidence interval (CI): 13.50–15.09], 4.81% [4.32–5.29], 5.19% [4.71–5.67], 5.79% [5.29–6.28] and 7.72% [7.17–8.27], respectively.

Among a total of 750 individuals diagnosed as cases of CHD (398 in men), 117 (15.6%), 453 (60.4%), and 317 (42.3%) individuals had a history of myocardial infarction (MI), cardiac procedure, and unstable angina, respectively. The age-standardized prevalence of CHD for the Tehranian population was 7.71% [7.18–8.24] in the total population, 8.62 [7.81–9.44] in men and 7.19 [6.46–7.93] in women. Moreover, among diabetic participants, the age-standardized prevalence of CHD was 13.10 [9.83–16.38] in men and 10.67 [8.90–12.44] in women, significantly higher than corresponding values for NFG/NGT and prediabetic groups.

Across six levels of glycemic status, CHD was associated with IFG/IGT [odds ratio (OR) and 95% CI: 1.38 (1.01–1.89)], NDM [1.83 (1.40–2.41)], and KDM [2.83 (2.26–3.55)] groups, in the age- and sex-adjusted model. Furthermore, in the full-adjusted model, only NDM and KDM status remained to be associated with the presence of CHD by ORs of 1.40 (1.06–1.86) for NDM and 1.91 (1.51–2.43) for KDM.

**Conclusion:**

The high prevalence of CHD, especially among diabetic populations, necessitates the urgent implementation of behavioral interventions in the Tehranian population, according to evidence-based guidelines for the clinical management of diabetic patients.

## Background

Coronary heart disease (CHD) is one of the most common causes of death worldwide [1, 2]. Its global fatality rate increased from 7.3 million in 2007 to 8.93 million deaths in 2017 [1]. We previously reported that the overall prevalence of CHD was 21.8% (22.3% among women and 18.8% among men) in 1991–2001 among residents of Tehran, as a metropolitan city [3]. Moreover, in Tehran, the incidence rate of CHD was 10.5 and 6.1 per 1000 person-years among men and women, respectively [4]. Importantly, among Tehranian adults aged ≥30 years, over 40% of mortality is attributed to cardiovascular disease (CVD) [5].

Type 2 diabetes mellitus (T2DM) is a major leading factor for CHD and its mortality [6, 7]. In addition to the well-known relationship between diabetes and CVD, it has also been shown that prediabetes status could lead to CHD and CVD [8, 9]. Based on national studies in 2011, about 11.4 and 14.6% of Iranian adults had diabetes mellitus (DM) and impaired fasting glucose (IFG), respectively. Furthermore, there was an alarming increase of 35.1% in the prevalence of DM from 2005 to 2011 [10]. A prediabetes tsunami (included both impaired glucose tolerance (IGT) and IFG) was also reported among a Tehranian population, with ≥4% of adult individuals developing prediabetes each year [11].

The current study aims to report the population-based prevalence of CHD among Tehranian adults, aged ≥30 years, according to their glycemic status in phase IV (2008–2011) of the oldest cohort study in the Middle East and North Africa (MENA) region, the Tehran Lipid and Glucose Study (TLGS) [12].

## Methods

### Study design and population study

The current study was performed within the framework of the TLGS, which is an ongoing cohort study being conducted on a representative sample of Tehranian citizens. The TLGS have the aim of determining the epidemiological aspects of non-communicable diseases (NCDs) and their risk factors. The TLGS also intended to prevent NCDs by developing healthier lifestyles. Further details for the TLGS have been described before [12]. Briefly, after the first baseline examination (1999–2001), participants were followed-up until 2011. For this study, 8400 individuals aged ≥30 years were enrolled from phase IV of TLGS (2008–2011). Firstly, we excluded 497 individuals whose glycemic status was not differentiable for us. Secondly, we excluded 177 subjects with missing data on covariates, including body mass index (BMI), total cholesterol (TC), high-density lipoprotein cholesterol (HDL-C), systolic blood pressure (SBP), diastolic blood pressure (DBP), family history of CVD, and smoking status (overlap features between numbers considered). Finally, due to the lack of information on the outcome (CHD) assessment, eight individuals were excluded, and 7718 participants remained eligible for analysis of the current study.

### Clinical and laboratory measurements

Using pretested questionnaires, an interviewer gathered data that included demographic data, smoking status, education level, drug history, past medical history, and family history. Details of blood pressure (BP) and anthropometric parameters measurements in the TLGS setting have been published previously [8]. After over 12 h of fasting, blood samples were drawn between 07:00 AM and 09:00 AM and then analyzed on the same day. Apart from those who had on glucose-lowering medications, a standard oral glucose tolerance test with 75 g glucose was done for all participants. Fasting plasma glucose (FPG) and 2-h post-challenge plasma glucose (2 h-PCPG) were measured by enzymatic colorimetric glucose oxidase method, both inter-and intra-assay coefficient of variations were < 2.2%. More details of laboratory measurements have been published elsewhere [8].

### Definition of terms

Participants were categorized into different groups as follows: Normal fasting glucose (NFG)/normal glucose tolerance (NGT), FPG < 5.6 and 2 h-PCPG < 7.7 mmol/L; isolated impaired fasting glucose (iIFG), 5.6 ≤ FPG < 7 and 2 h-PCPG < 7.7 mmol/L; isolated impaired glucose tolerance (iIGT), 7.7 ≤ 2 h-PG < 11.1 and FPG < 5.6 mmol/L; combined IFG and IGT (IFG/IGT), 5.6 ≤ FPG < 7 and 7.7 ≤ 2 h-PCPG < 11.1 mmol/L [9]. Moreover, in the present study, prediabetes status was defined as the presence of IFG or IGT. Finally, newly diagnosed diabetes mellitus (NDM) was defined as FPG ≥ 7.0 or 2 h-PCPG ≥11.1 mmol/L among those participants were not on glucose-lowering medications and known diabetes mellitus (KDM) as subjects with positive history of taking any glucose lowering medications. Having TC ≥ 5.2 mmol/L or using lipid-lowering medications defined as hypercholesterolemia. Low HDL-C was defined as HDL-C < 1.036 mmol/L for men and < 1.295 mmol/L for women, or taking lipid-lowering medications. Based on the seventh report of the Joint National Committee on prevention, detection, evaluation, and treatment of high blood pressure (the JNC 7 report) [13], hypertension was considered as either of having SBP ≥140 mmHg or DBP ≥90 mmHg or the usage of any anti-hypertensive medications. Smoking status was categorized into three levels, including current, past, and never smoker. Education levels were classified as < 6 years (reference group), 6–12 years, and > 12 years. By the Modifiable Activity Questionnaire (MAQ), which judged all types of activities [8], physical activity was evaluated. Low physical activity (inactive person) was defined as not achieving a minimum score of 600 MET (metabolic equivalent task)-minutes per week [14]. If there was at least one history of CHD/stroke in a male first-degree relative aged < 55 years or in a female first-degree relative aged < 65 years, the family history of premature CVD is considered positive.

### Definition of CHD

Details of the collection of outcome data have been reported elsewhere [8]. To summarize, each individual was under continuous surveillance for any medical outcome leading to hospitalization. As a part of the cohort data collection, a trained nurse called all participants annually and recorded any medical events experienced during the last year. A trained physician followed-up any reported event by a home visit for medical data gathering. Collected data were then evaluated by a consulting committee, the outcome committee, included a principal investigator, an internist, an endocrinologist, a cardiologist, an epidemiologist, and the physician that collected the outcome data. Every confirmed event was considered as a NCD outcome based on ICD-10 criteria [8, 15]. In this study, CHD was selected from ICD-10 rubric I20-I25. CHD cases included [15–18]:

1. **Myocardial infarction (MI),** included a) definite MI diagnosed by diagnostic electrocardiogram (ECG) and biomarkers (including CK, CK-MB, CK-MBm, troponin (cTn), and myoglobin), b) probable MI distinguished by positive ECG findings plus cardiac symptoms or signs and biomarkers showing negative or equivocal results.
2. **Cardiac procedure,** defined as a) angiography proven CHD with a result of ≥ 50% stenosis in at least one major coronary vessel, b) history of angioplasty or bypass surgery.
3. **Unstable angina pectoris,** who developed new cardiac symptoms or showed changing symptom patterns and positive ECG findings with normal biomarkers and admitted to coronary care unit (CCU).

### Statistics

Baseline characteristics are presented as means ± standard deviations (SD), median (interquartile range), and number (frequency) as appropriate. ANOVA and Kruskal-Wallis tests were used for comparison of means and medians, respectively. Chi-squared test was applied for comparison of frequencies.

The crude and age-standardized prevalence (95% confidence interval: CI) were calculated for all glycemic status, including NFG/NGT, iIFG, iIGT, IFG/IGT, NDM, and KDM. Regarding differences in the age distributions between the TLGS population from 2008 to 2011 and the Iranian census 2010 (supplementary Table 1), especially in the 30–39-year age-group and those aged ≥70 years, the age-standardized prevalence was reported, using the Iranian (Tehran province) census 2010.

We also examined the association of different glycemic status with the prevalence of CHD. Using logistic regression analyses, odds ratios (ORs) for this association were calculated in 3 levels of adjustment: 1) without adjustment (crude OR); 2) age and sex adjustment; 3) full adjustment (adjusted for age, sex, BMI, hypercholesterolemia, low HDL-C, hypertension, family history of premature CVD, and smoking status).

Statistical analyses were done using STATA version 14. *P*-values < 0.05 were considered to be statistically significant.

## Results

The study sample included 7718 participants (men = 3427) aged ≥30 years [mean age (SD) 50.1 (13.2) years]. Sex-specific baseline characteristics across glycemic categories are shown in Table 1. Generally, in comparison with the prediabetes and DM groups, participants with NFG/NGT had better cardiometabolic risk profiles, including age, BMI, waist to hip ratio, triglycerides, TC (only among women), SBP, and DBP. Furthermore, compared to the prediabetes and DM groups, participants with NFG/NGT had a better education status, lower prevalence of low physical activity (among women), and lower frequency of lipid-lowering and anti-hypertensive medications usage. Moreover, for most above-mentioned factors, prediabetic participants were ranked between NFG/NGT and DM groups.

**Table 1:** Baseline characteristics of participants across glycemic categories: Tehran Lipid and Glucose Study (phase IV: 2008–2011)

	Men				Women			
	NFG/NGT	Prediabetes (IFG or IGT)	DM	***P***-value*	NFG/NGT	Prediabetes (IFG or IGT)	DM	***P***-value*
Number of participants	1891	1000	536		2531	1040	720	
Continuous variables, Mean ± SD								
Age (year)	47.2 ± 12.8	53.6 ± 13.7	59.5 ± 12.6	< 0.001	45.2 ± 11.5	52.8 ± 11.9	58.7 ± 10.7	< 0.001
BMI (kg/m^2^)	26.6 ± 4.0	28.0 ± 4.1	28.1 ± 4.1	< 0.001	28.5 ± 4.7	31.4 ± 18	31.3 ± 5.6	< 0.001
Waist to hip ratio × 100	95.9 ± 6.3	98.6 ± 5.7	100.6 ± 5.7	< 0.001	90.7 ± 7.6	95.2 ± 7.4	99.2 ± 6.8	< 0.001
SBP (mmHg)	117.1 ± 15.6	124.0 ± 18.3	130 ± 19.2	< 0.001	111.9 ± 16.2	122.2 ± 19.5	129.8 ± 22.0	< 0.001
DBP (mmHg)	78.9 ± 10.5	80.8 ± 10.8	82.3 ± 11.0	< 0.001	74.7 ± 10.4	78.4 ± 10.8	79.8 ± 11.6	< 0.001
Total cholesterol (mmol/L)	4.9 ± 0.9	5.0 ± 1.0	4.8 ± 1.0	< 0.001	5.0 ± 0.9	5.4 ± 1.0	5.3 ± 1.2	< 0.001
HDL-C (mmol/L)	1.1 ± 0.2	1.1 ± 0.2	1.1 ± 0.2	0.109	1.3 ± 0.3	1.3 ± 0.3	1.2 ± 0.3	< 0.001
Triglyceride (mmol/L)	1.5 (1.1–2.1)	1.7 (1.2–2.3)	1.8 (1.3–2.5)	< 0.001	1.2 (0.9–1.7)	1.6 (1.2–2.2)	1.9 (1.4–2.8)	< 0.001
Categorical variables, n (%)								
Smoking status				< 0.001				0.382
- Never	1055 (55.8%)	578 (57.8%)	303 (56.5%)		2403 (94.9%)	994 (95.6%)	690 (96.0%)	
- Past	345 (18.2%)	227 (22.7%)	140 (26.1%)		45 (1.8%)	16 (1.5%)	15 (2.1%)	
- Current	491 (26.0%)	195 (19.5%)	93 (17.4%)		83 (3.3%)	30 (2.9%)	14 (1.9%)	
Education level				< 0.001				< 0.001
- < 6 years	283 (15.0%)	256 (25.6%)	198 (36.9%)		578 (22.8%)	448 (43.1%)	450 (62.6%)	
- 6–12 years	1042 (55.1%)	524 (52.4%)	255 (47.6%)		1405 (55.5%)	491 (47.2%)	232 (32.3%)	
- > 12 years	566 (29.9%)	220 (22.0%)	83 (15.5%)		548 (21.7%)	101 (9.7%)	37 (5.1%)	
Low physical activity, yes	723 (39.6%)	401 (43.3%)	203 (41.6%)	0.169	620 (24.6%)	272 (26.6%)	259 (37.6%)	< 0.001
Family history of premature CVD, yes	78 (4.1%)	43 (4.3%)	28 (5.2%)	0.543	160 (6.3%)	81 (7.8%)	47 (6.5%)	0.276
Lipid-lowering medication, yes	80 (4.2%)	96 (9.6%)	129 (24.1%)	< 0.001	129 (5.1%)	131 (12.6%)	262 (36.4%)	< 0.001
Anti-hypertensive medication, yes	126 (6.7%)	121 (12.1%)	148 (27.6%)	< 0.001	194 (7.7%)	215 (20.7%)	317 (44.1%)	< 0.001

Values are shown as Mean ± SD and number (%), for continuous and categorical variables, respectively; Triglycerides are given as median (interquartile range) due to skewed distribution

*BMI* Body mass index, *SBP* Systolic blood pressure, *DBP* Diastolic blood pressure, *HDL-C* High density lipoprotein cholesterol, *CVD* Cardiovascular disease, *NFG* Normal fasting glucose, *NGT* Normal glucose tolerance, *IFG* Impaired fasting glucose, *IGT* Impaired glucose tolerance, *DM* Diabetes mellitus

*The comparison *p*-value between groups was calculated using ANOVA test for normal continues variables, chi-square test for categorical variables and kruskal wallis test for skewed variables

**Fig. 1:**
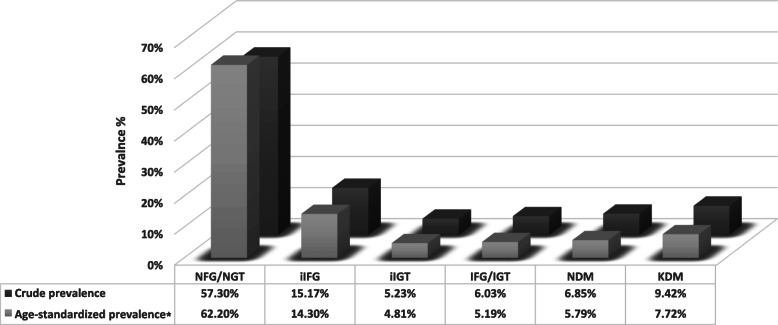
Prevalence of different glycemic status: Tehran Lipid and Glucose Study (phase IV: 2008–2011). * Age-standardized prevalence is calculated based on Iranian population distribution data from the National Consensus Bureau for Tehran province (2010). NFG: normal fasting glucose; NGT: normal glucose tolerance; iIFG: isolated impaired fasting glucose; iIGT: isolated impaired glucose tolerance; IFG/IGT: both impaired fasting glucose and impaired glucose tolerance; NDM: newly diagnosed diabetes mellitus; KDM: known diabetes mellitus

Figure 1 shows the crude and age-standardized prevalence of different glycemic status. Among our total population, the age-standardized prevalence of iIFG, iIGT, IFG/IGT, NDM, and KDM were 14.30% (13.50–15.09), 4.81% (4.32–5.29), 5.19% (4.71–5.67), 5.79% (5.29–6.28), and 7.72% (7.17–8.27), respectively.

**Fig. 2:**
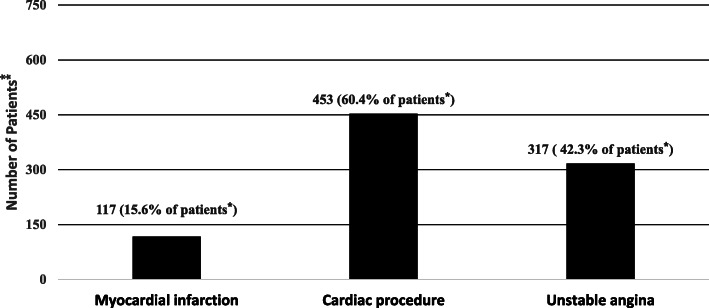
Number of patients across different types of coronary heart disease (CHD): Tehran Lipid and Glucose Study (phase IV: 2008–2011). * Percentage of each type were calculated only among 750 patients with positive coronary heart disease in our data set. ** The total number of different type of CHD is over 750 (100%), considering that patients might have more than one type of CHD. CHD events included cases of: (1) Myocardial infarction (MI), included a) definite MI diagnosed by diagnostic electrocardiogram (ECG) and biomarkers (including CK, CK-MB, CK-MBm, troponin (cTn), and myoglobin), b) probable MI distinguished by positive ECG findings plus cardiac symptoms or signs and biomarkers showing negative or equivocal results. (2) Cardiac procedure, defined as a) angiography proven CHD with a result of ≥ 50% stenosis in at least one major coronary vessel, b) history of angioplasty or bypass surgery. (3) Unstable angina pectoris, who developed new cardiac symptoms or showed changing symptom patterns and positive ECG findings with normal biomarkers and admitted to coronary care unit (CCU)

Seven hundred fifty individuals were diagnosed as cases of CHD. The crude and age-standardized prevalence of CHD for the Tehranian population were 9.72% (95% CI: 9.06–10.38) and 7.71% (7.18–8.24), respectively. As is illustrated in Fig. 2, from a total of 750 patients with CHD in this study, 117 (15.6%), 453 (60.4%), and 317 (42.3%) individuals had a history of MI (definite and probable MI), cardiac procedure, and unstable angina, respectively. It should be noted that the total number of different types of CHD is over 750 (100%), considering that patients might have more than one type of CHD.

The sex-specific prevalence of CHD across glycemic categories is shown in Table 2. Among the total age group, the crude prevalence of CHD was 11.61 (10.54–12.69) in men, which was significantly higher than the corresponding number among women. After age standardization, the prevalence decreased to 8.62 (7.81–9.44) in men and 7.19 (6.46–7.93) in women. Among diabetic participants, the age-standardized prevalence of CHD was 13.10 (9.83–16.38) in men and 10.67 (8.90–12.44) in women, significantly higher than corresponding values for prediabetes and NFG/NGT groups.

**Table 2:** Prevalence of coronary heart diseases across glycemic categories, by gender: Tehran Lipid and Glucose Study (phase IV: 2008–2011)

	Men			Women		
	Case/Total	Crude prevalence % (95% CI)	Age-standardized prevalence ^**a**^ % (95% CI)	Case/Total	Crude prevalence % (95% CI)	Age-standardized prevalence ^**a**^ % (95% CI)
**NFG/NGT**	139/1891	7.35 (6.17–8.53)	7.28 (6.06–8.49)	108/2531	4.27 (3.48–5.05)	5.71 (4.62–6.79)
**Prediabetes (IFG or IGT)**	126/1000	12.60 (10.54–14.66)	7.95 (6.55–9.36)	88/1040	8.46 (6.77–10.15)	6.62 (5.06–8.19)
**DM**	133/536	24.81 (21.15–28.47)	13.10 (9.83–16.38)	156/720	21.67 (18.65–24.68)	10.67 (8.90–12.44)
**Total**	398/3427	11.61 (10.54–12.69)	8.62 (7.81–9.44)	352/4291	8.20 (7.38–9.02)	7.19 (6.46–7.93)

*NFG* Normal fasting glucose, *NGT* Normal glucose tolerance, *IFG* Impaired fasting glucose, *IGT* Impaired glucose tolerance, *DM* Diabetes mellitus, *CI* Confidence interval

^a^Age-standardized prevalence is calculated based on Iranian population distribution data from the National Consensus Bureau for Tehran province (2010)

The prevalence and ORs of CHD across NFG/NGT (as reference group), iIFG, iIGT, combined IFG and IGT, NDM, and KDM groups are presented in Table 3. KDM and NFG/NGT groups had the highest and lowest crude prevalence of CHD, respectively. Moreover, among prediabetic groups, the prevalence of CHD was tended to be more prominent in the combined IFG and IGT. The age-standardized prevalence of CHD was estimated to be 6.39 (5.59–7.19), 6.52 (5.23–7.82), 7.00 (4.73–9.27), 8.04 (5.40–10.67), 8.74 (7.08–10.40), and 14.26 (10.73–17.79) among NFG/NGT, iIFG, iIGT, combined IFG and IGT, NDM, and KDM groups, respectively. After adjustment for age and sex, CHD was more likely to be associated with combined IFG and IGT, NDM, and KDM groups. Furthermore, in the full-adjusted model, NDM and KDM status remained to be significantly associated with the presence of CHD by ORs of 1.40 (1.06–1.86) for NDM and 1.91 (1.51–2.43) for KDM.

**Table 3:** Prevalence and odds ratio of coronary heart diseases across glycemic categories: Tehran Lipid and Glucose Study (phase IV: 2008–2011)

	Case/Total	Crude prevalence % (95% CI)	Age-standardized prevalence ^**a**^ % (95% CI)	Crude odds ratio (95% CI)	Age and sex adjusted odds ratio (95% CI)	Full-adjusted odds ratio ^**b**^ (95% CI)
**NFG/NGT**	247/4422	5.59 (4.91–6.26)	6.39 (5.59–7.19)	Reference	Reference	Reference
**iIFG**	105/1171	8.97 (7.33–10.60)	6.52 (5.23–7.82)	1.67 (1.31–2.11)	1.13 (0.88–1.45)	0.99 (0.76–1.27)
**iIGT**	45/404	11.14 (8.07–14.21)	7.00 (4.73–9.27)	2.12 (1.52–2.96)	1.17 (0.82–1.67)	0.95 (0.66–1.38)
**IFG/IGT**	64/465	13.76 (10.63–16.90)	8.04 (5.40–10.67)	2.70 (2.01–3.62)	1.38 (1.01–1.89)	1.07 (0.78–1.47)
**NDM**	98/529	18.53 (15.21–21.84)	8.74 (7.08–10.40)	3.84 (2.98–4.96)	1.83 (1.40–2.41)	1.40 (1.06–1.86)
**KDM**	191/727	26.27 (23.07–29.47)	14.26 (10.73–17.79)	6.02 (4.89–7.43)	2.83 (2.26–3.55)	1.91 (1.51–2.43)
**Total**	750/7718	9.72 (9.06–10.38)	7.71 (7.18–8.24)	_	_	_

*NFG* Normal fasting glucose, *NGT* Normal glucose tolerance, *iIFG* Isolated impaired fasting glucose, *iIGT* Isolated impaired glucose tolerance, *IFG/IGT* Both impaired fasting glucose and impaired glucose tolerance, *NDM* Newly diagnosed diabetes mellitus, *KDM* Known diabetes mellitus, *CI* Confidence interval

^a^Age-standardized prevalence is calculated based on Iranian population distribution data from the National Consensus Bureau for Tehran province (2010)

^b^Adjusted for age, sex, BMI, hypercholesterolemia, low high-density lipoprotein cholesterol, hypertension, family history of premature cardiovascular disease, and smoking status

## Discussion

In this population-based study conducted in 2008–2011, 14.30, 4.81, 5.19, 5.79, and 7.72% of Tehranian residents were found to be iIFG, iIGT, IFG/IGT, NDM, and KDM, respectively. The age-standardized prevalence of CHD was about 7.7% among Tehranian residents. Generally, compared to women, the prevalence of CHD was found to be in higher ranges among men. Additionally, the age-standardized prevalence of CHD among diabetic participants was reported to be 13.1% in men and 10.7% in women. After age and sex adjustment, compared to the NFG/NGT group, the presence of IFG/IGT, NDM, and KDM were significantly associated with higher prevalence of CHD. The associations were significant for NDM and KDM, even in the full-adjusted model.

Based on the current study, about 40% of Tehranian adults were in prediabetes or diabetes status in 2008–2011, which was higher than our previous finding in 1990–2001 (about 30%) [19]. In 2005–2011, national studies found a 35% increase in DM prevalence; the prevalence reached 11.4% in 2011. Importantly, in this period, DM awareness improved, and the nation-wide prevalence of NDM decreased from 45.7 to 24.7% [10]. Additionally, the researchers found that about 14.6% of Iranian adults (15.4% among urban residents) were in IFG status in 2011 [10], which was comparable to our study.

In our previous study, using self-reported history of CHD, Rose angina, and ECG-defined ischemia for CHD definition, a 21.8% prevalence of CHD was reported for Tehranian adults in 1999–2001 [3]. The differences between the prior study and the current study might be attributable to the following factors. Firstly, in our previous report, the silent ischemia and positive history of Rose angina were considered as cases of CHD; however, we did not consider these soft criteria of CHD in the current study. Secondly, for the history of CHD, in contrast to our original report, it was considered positive only when its hospital records were provided and then adjudicated by the outcome committee. Hence, in the current study, using the solid criteria for the definition of CHD led to underestimations of the prevalence of CHD.

It is important to note that due to different diagnostic criteria for CHD and different baseline characteristics of the population study, comparing our results with other population-based studies is somewhat difficult. Abbasi et al. reported that among an Iranian population aged over 20 years, the national prevalence of self-reported CHD was 5.3% (5.6% among urban residents) in 2011 [20]; their values for the prevalence of self-reported CHD were significantly lower than our reports. Compared to developed countries, data from the Quebec Integrated Chronic Disease Surveillance System (QICDSS) showed that the age-standardized prevalence of CHD (CHD death not included) was 7.7% among a Canadian adult population in 2009/2010 [21], which was comparable to ours. The American Heart Association (AHA) reported that the prevalence of total CHD was 6.7% among US adults aged ≥20 years (7.4% for men and 6.2% for women) [22]. For the United Kingdom (UK), data from the Quality and Outcomes Framework (QOF) indicated that the prevalence of CHD remained constant at about 3% in England and 4% in Scotland, Wales, and Northern Ireland between 2004/2005 and 2014/2015 [23]. Additionally, the prevalence of CHD varied from 2 to 4% in national studies of India [24]. Furthermore, in Saudi Arabia, as a Middle Eastern country, the age-standardized prevalence of CHD was reported to be 5.9 and 4.4% among male and female adults, respectively [25]. Generally, it seems that the estimated prevalence of CHD among the Tehranian population is higher than corresponding figures in US [22], UK [23], India [24] and, Saudi Arabia [25], an issue previously addressed in 2015 by Zhu et al. [26]. As we reported previously, modifiable risk factors (diabetes, hypertension, smoking, and dyslipidemia) had population attributable fraction of 36.6 and 50.2% for incident CHD among the male and female Tehranian populations, respectively [4]. Other reasons that might justify the high prevalence of CHD among the Tehranian population are related to the impact of air pollution [27, 28] and stress [29], which are common in Tehran.

Focusing on diabetes status, from a national study on Iranian diabetic patients aged ≥18 years, it was reported that the crude prevalence of CHD was 25.1% for men and 23.2% for women in 2016 [30], which were comparable to our findings (24.81% for men and 21.67% for women). Among diabetic populations of other countries, the age-standardized prevalence of CHD was found to be 4.43 and 4.76% among Chinese men and women with T2DM, respectively [31]. Moreover, the prevalence of CHD among Thai patients with diabetes was 3.54% in 2013 [32]. Additionally, among Swedish diabetic patients aged 45–74 years, the crude prevalence of CHD was reported to be 24.9% for men and 18.0% for women [33]. In addition, a significant racial difference was reported in the prevalence of CHD between White and African diabetic patients in a hospital-based study [34]. It has been suggested that there is also a racial susceptibility for CHD among diabetic patients, which could make Iranian diabetic patients more prone to developing CHD, compared to Asian, African, and European ethnicities. Furthermore, although CVD risk factors among Iranian diabetic populations have been controlled to some degree, during recent years, most diabetic participants still have uncontrolled CVD risk factors [35], which could also lead to a high prevalence of CHD among our diabetic population.

In the current study, as expected, the significantly highest prevalence of CHD was found among participants with KDM. We also found that compared to NFG/NGT group, the presence of NDM status was associated with CHD [adjusted OR = 1.40 (1.06–1.86)]. We have previously reported that during a 7.6 years follow-up, Tehranian adults with NDM exhibited a CHD risk comparable to non-DM with a prior CHD [36]. In a cohort study, conducted on 271,174 participants with T2DM who were in the Swedish National Diabetes Register, it was shown that patients with T2DM who had five risk factors within the target range, appeared to have little or no excess risk of MI, in comparison with the general population [37]. Hence, we suppose that tight control of all CVD risk factors among the Iranian diabetic population should be considered in health policies to halt the increasing burden of CVD events. Regarding prediabetes status, a significant association was found between combined IFG/IGT and CHD in the sex- and age-adjusted model. Among a non-diabetic population, it was also reported that participants with combined IFG and IGT had a higher prevalence of significant CHD and higher severity of disease in their angiographic results; however, there were no significant differences among subjects with NGT, i-IFG, and i-IGT [38].

Our study has its strengths in adjudicated cases of CHD by an outcome committee and the determination of CHD prevalence across different glycemic status, using the glucose challenge test. Several limitations need to be acknowledged. First, our study shows an optimistic picture of CHD prevalence among our population since the inclusion of subjects in an ongoing study can improve the level of attention paid to controlling their health risks (cohort effect). Therefore, the burden of CHD might be much higher in the context of the community. Second, this investigation was conducted among residents of Tehran as a metropolitan city. Hence, our results might not be generalizable to rural zones.

## Conclusion

The high prevalence of CHD, especially among diabetic populations, necessitates urgent behavioral intervention to be aimed at halting obesity tsunami [39], hypertension [40], and physical inactivity [41] among the Tehranian population, according to evidence-based guidelines for the clinical management of diabetic patients. Last but not least, the impact of environmental and psychosocial factors on CHD in Tehranians should be investigated in future studies.

## Supplementary information

**Additional file 1: Table S1.** Age distribution of the Iranian population for Tehran province (2010) and the sample population of Tehran lipid and glucose study (2008–2011).

## Data Availability

The datasets used and analyzed during the current study are available from the corresponding author on reasonable request.

## Abbreviations

CHD: Coronary Heart Disease
CVD: Cardiovascular Disease
T2DM: Type 2 Diabetes Mellitus
DM: Diabetes Mellitus
IFG: Impaired Fasting Glucose
IGT: Impaired Glucose Tolerance
MI: Myocardial Infarction
ECG: Electrocardiogram
MENA: Middle East and North Africa
TLGS: Tehran Lipid and Glucose Study
NCD: Non-communicable Disease
BP: Blood Pressure
BMI: Body Mass Index
TC: Total Cholesterol
HDL-C: High Density Lipoprotein Cholesterol
SBP: Systolic Blood Pressure
DBP: Diastolic Blood Pressure
FPG: Fasting Plasma Glucose
2 h-PCPG: 2-h Post Challenge Plasma Glucose
NFG: Normal Fasting Glucose
NGT: Normal Glucose Tolerance
iIFG: Isolated Impaired Fasting Glucose
iIGT: Isolated Impaired Glucose Tolerance
IFG/IGT: Combined IFG and IGT
NDM: Newly Diagnosed Diabetes
KDM: Known Diabetes Mellitus
MAQ: Modifiable Activity Questionnaire
CCU: Coronary Care Unit
SD: Standard Deviations
CI: Confidence Interval
QICDSS: Quebec Integrated Chronic Disease Surveillance System
UK: United Kingdom
QOF: Quality and Outcomes Framework
