# Supplementary material for: Sex-specific prevalence of coronary heart disease among Tehranian adult population across different glycemic status: Tehran lipid and glucose study, 2008–2011
Source: BMC Public Health. 2020 Oct 6;20:1510. doi: 10.1186/s12889-020-09595-4 (PMC7539419; doi:10.1186/s12889-020-09595-4)
Supplement: Supplementary file 1 — Additional file 1: Table S1. Age distribution of the Iranian population for Tehran province (2010) and the sample population of Tehran lipid and glucose study (2008–2011). [file 12889_2020_9595_MOESM1_ESM.docx]

| **Supplementary table 1.** Age distribution of the Iranian population for Tehran province(2010) and the sample population of Tehran lipid and glucose study (2008-2011). | | | | | | | | | | | |
| --- | --- | --- | --- | --- | --- | --- | --- | --- | --- | --- | --- |
|  | Men | | |  | Women | | |  | Whole Population | | |
| Age (yrs) | Population | Sample | Sampling fraction |  | Population | Sample | Sampling fraction |  | Population | Sample | Sampling fraction |
| 30-39 | 1410634 | 822 | 0.058272 |  | 1411745 | 1123 | 0.079547 |  | 2822379 | 1945 | 0.068913 |
| 40-49 | 954218 | 963 | 0.100920 |  | 914846 | 1196 | 0.130732 |  | 1869064 | 2159 | 0.115512 |
| 50-59 | 692185 | 683 | 0.098673 |  | 697876 | 979 | 0.140283 |  | 1390061 | 1653 | 0.118916 |
| 60-69 | 404488 | 511 | 0.126333 |  | 409817 | 668 | 0.163000 |  | 814305 | 1179 | 0.144786 |
| 70-79 | 196176 | 375 | 0.191155 |  | 196531 | 299 | 0.152139 |  | 392707 | 674 | 0.171629 |
| ≥ 80 | 89475 | 73 | 0.081587 |  | 89058 | 35 | 0.039300 |  | 178533 | 108 | 0.060493 |
| For sample fraction, the ratio numbers are multiplied by 100. Standard age distribution of the Iranian population are based on data from the National Consensus Bureau for Tehran province (2010) | | | | | | | | | | | |
